# Women's Experience of Depressive Symptoms While Working From Home During the COVID-19 Pandemic: Evidence From an International Web Survey

**DOI:** 10.3389/fsoc.2022.763088

**Published:** 2022-04-08

**Authors:** Emily Burn, Giulia Tattarini, Iestyn Williams, Linda Lombi, Nicola Kay Gale

**Affiliations:** ^1^Health Services Management Centre, University of Birmingham, Birmingham, United Kingdom; ^2^Social Science Research Center Berlin, Berlin, Germany; ^3^Department of Sociology and Social Research, University of Trento, Trento, Italy; ^4^Department of Sociology, Università Cattolica del Sacro Cuore, Milan, Italy

**Keywords:** COVID-19, mental health, depression, women, home-working

## Abstract

The COVID-19 pandemic led to significant changes in workplace practices as social distancing requirements meant that people were asked to work from home where possible to avoid unnecessary contact. Concerns have been raised about the effects of the pandemic on mental health and, in particular, the effects of social distancing on employed women's mental health. In this study, we explore the experiences of working women during the initial stages of the COVID-19 pandemic and explore the factors that may be associated with women experiencing the symptoms of depression. Findings from a cross-sectional survey of European working women (across five countries: France, Italy, Poland, Sweden, and the UK) conducted between March and July 2020 are reported. The data are analyzed using linear regression and mediation analysis. For women, working from home was associated with higher prevalence of the symptoms of depression compared to traveling to a workplace. The study also considers the mechanisms that may explain a relationship between working from home and depressive symptoms. Maintaining contact with people face-to-face and participating in exercise were both significant protective factors against experiencing symptoms of depression during a period of social distancing.

## Introduction

The early stages of the COVID-19 pandemic led to requirements for people to practice “social distancing”, i.e., to reduce the level of face-to-face contact they have with other people. Related to this there were significant changes in workplace practices, with people asked to work from home where they could do so to avoid unnecessary contact with colleagues or clients. In many cases, workers have had to balance this with additional caring responsibilities, such as supporting children with home schooling. Concerns have been raised about the effects of the pandemic on mental health and, in particular, women's mental health (Almeida et al., 2020; Thibaut and van Wijngaarden-Cremers, 2020). At the start of the COVID-19 pandemic, a vast number of people started to work from home for the first time. There have been some studies which have explored women's experience of working from home during the COVID-19 pandemic (Möhring et al., 2021; Zoch et al., 2021), however, to date, there has not been a focused exploration of women's experience of working from home and mental health.

In this paper, we explore the association between working from home and women's experience of the symptoms of depression at the start of the pandemic. We found that women working from home did experience higher levels of depressive symptoms than women working outside of the home. Our paper explores possible factors (social contact, exercise, and caring for children) that may contribute to an increased prevalence of depression for women who worked from home.

## COVID-19 and the Effects of Social Distancing on Depression: Previous Findings and Theoretical Perspectives

The COVID-19 pandemic precipitated the introduction of social distancing policies by governments across many countries, with people required to severely curtail their level of social contact with others outside of their household. Studies confirm that social distancing in response to the first wave of COVID-19 coincided with worsening mental health across multiple countries (Alzueta et al., 2020; Zhao et al., 2020; Cecchini et al., 2021). Social distancing policies have also resulted in an increase in the number of people working from home. For example, in the UK, 5.1% of employed people reported working mainly from home in 2019 (Office for National Statistics, 2020b), and this increased to 46.6% of employed people doing “some” work from home in April 2020, the first full month of social distancing measures (Office for National Statistics, 2020a). Anderson and Kelliher (2020) highlight that working from home *can* lead to an increased sense of autonomy and greater choice over working arrangements, but question whether these benefits remain when working from home is enforced, as is the case during the COVID-19 pandemic. However, Möhring et al. (2021) find that, in Germany, working from home was not significantly associated with women's levels of satisfaction with work or satisfaction with family life. Given the increase in the number of people working from home during the early stage of the COVID-19 pandemic, it is of interest to explore people's experience of working from home. In this article, we focus on the experiences of women in paid employment in Europe during COVID-19.

Women in general have been identified as experiencing higher levels of mental ill-health compared to men (NHS Digital, 2014) and they have continued to experience higher levels of mental ill-health during the first wave of COVID-19 (Banks and Xiaowei, 2020; Gray et al., 2020; Pieh et al., 2020; Maffly-Kipp et al., 2021; Paudel, 2021; Prati, 2021). Although there will be variation in women's experiences of the pandemic, particularly along lines of class/socio-economic status, “race”/ethnicity, and other demographic factors, COVID-19 has disrupted working patterns across society in a way previously unthinkable. At the start of the pandemic, a minority of people were classed as “essential” workers, who are required to continue operating in their usual place of work. There was not a uniform definition of the roles considered to be “essential” workers. Furthermore, there is some disagreement in the literature about whether women are more or less likely to be essential workers (Alon et al., 2020; Hupkau and Petrongolo, 2020). Either way, while essential workers might be expected to experience higher levels of stress as a result of the combination of increased work-related demands during the early stages of the pandemic, and concern about being exposed to the virus (Carli, 2020), there is no evidence that this is the case. Being an essential worker has been linked to significantly lower levels of depression during the first stages of the COVID-19 pandemic (White and Van Der Boor, 2020). At the other end of the spectrum, many people have lost employment or were furloughed, i.e., been granted a (partially) paid leave of absence from their work, meaning that they were at home continuously during the early stages of the pandemic. The initial lockdowns due to the COVID-19 pandemic resulted in an increase in the amount of time people spent at home and, for women, this may have disproportionate effects on their risk of domestic violence (Van Gelder et al., 2020). For a large proportion of the population, social distancing regulations have meant that they have been required to “work from home” (also known as smart working or remote working), adapting their working practices accordingly, such as the use of video technology for meetings. Women, if not working within a sector closed due to social distancing, are more likely to have jobs that can be carried out from home (Hupkau and Petrongolo, 2020). Although this can offer some protection from job-loss (Adams-Prassl et al., 2020), the impact of these changed working practices on mental health is not clear.

In this article, we examine whether there is an association between working from home and women's mental health in European working women. Specifically, we consider whether women working from home experience a higher prevalence of the symptoms of depression compared to women who remain working at their usual place of work. We are not aware of any studies that have previously explored this. Our first research question was:

- *Did women working from home experience higher levels of the symptoms of depression during the COVID-19 pandemic compared to women working outside of the home?*

*Hypothesis 1. Women working from home experienced higher levels of the symptoms of depression compared to women working outside of the home*.

Our second research question was:

- *If the answer to 1. is yes, why might remote working have contributed to higher levels of the symptoms of depression for women?*

We investigated three potential mechanisms which might explain higher levels of depression: caring for children; decreased social contact; and exercise. The former focuses more on situational determinants, while the latter two are more focused on individual practices.

### Caring for Children

The first potential mechanism is that women working from home may experience increased pressures due to greater caring responsibilities. Findings on the effects of social distancing measures on the domestic division of labor in heterosexual couples have been relatively mixed. Some studies have identified an increase in men's participation in the home, where men are working remotely, are furloughed or have lost their jobs (Sevilla and Smith, 2020). Nevertheless, the amount of time women spent completing domestic tasks also increased (Chung et al., 2020; Farré et al., 2020), with men's increased domestic labor more likely to involve childcare rather than other “rather unpleasant” tasks such as cleaning or doing the laundry (Meraviglia and Dudka, 2021, p. 68). Furthermore, Del Boca et al. (2020) found that women's level of childcare did not appear to be affected by male partners' working arrangements, whereas men's levels of childcare did seem to be affected by female partner's working arrangements. This could be said to demonstrate the perceived primacy of childcare as primarily a woman's “role” and an underlying expectation that women balance childcare with work. Social distancing measures created particular challenges for working mothers who are likely to have to balance their employment with these additional domestic demands (Chung et al., 2020; Farré et al., 2020; Hipp and Bünning, 2021). Working mothers have been found to have experienced worse mental health during the pandemic (Benassi et al., 2020; Zamarro and Prados, 2020). Women working from home have been found to be more likely to be interrupted by their child's care needs compared to men and women have also been found to be more likely to schedule work around childcare demands (Anderson and Kelliher, 2020; Andrew et al., 2020). Zoch et al. (2021) finds a strong positive relationship between mothers in Germany working from home and exclusive maternal care of children, highlighting the dual demands remote working mothers may experience. The findings from these studies suggest that it is likely that mothers working from home will face more disruption to their working day compared to working mothers who travel to their usual workplace. As a result, mothers working from home are more likely to experience tensions as the opposing demands of family life and work increase and become more complex, creating inter-role conflict (Kahn et al., 1964). Feng and Savani (2020) found that, during COVID-19, women reported lower levels of perceived productivity and job satisfaction, and this was attributed to the additional domestic demands placed upon them. The additional stress and pressures resulting from increased childcare could potentially increase the likelihood of remote working mothers experiencing a higher prevalence of the symptoms of depression.

*Hypothesis 2. Remote working increases perceived conflict between work and caring for children which is associated with higher levels of the symptoms of depression for women*.

### Decreased Social Contact

The second potential mechanism that we consider is reduced levels of social contact. Social distancing has resulted in decreased social contact for most people, as social activities in leisure time and household mixing are restricted. There is a distinction to be made between the subjective feeling of loneliness and social isolation as someone may have limited social contacts and not experience loneliness. Although the factors influencing loneliness are complex, reduced social contact is associated with poorer mental health and higher levels of depression (Benke et al., 2020; Killgore et al., 2020) while higher perceived social connectedness is associated with lower levels of perceived stress (Nitschke et al., 2021). Reported loneliness has increased during periods of social distancing and women have reported higher levels of loneliness than men (Bu et al., 2020; Losada-Baltar et al., 2021; Niedzwiedz et al., 2021).

Given the association between perceived loneliness and depression, it may be expected that women working from home have reduced social contact and may be more likely to experience loneliness which could contribute to a greater prevalence of the symptoms of depression. Women traveling to their usual workplace are likely to maintain (at least) face-to-face contact with people they associate with in their employment. Previous studies have found that women typically have large and rich social networks and circles, when compared with men (Etheridge and Spantig, 2020). It is therefore possible that the experience of social distancing has been particularly disruptive for women's interpersonal ties which could lead to a reduction in mental wellbeing. Although their study focused on people aged 60 years and above in the US and the UK, Hu and Qian (2021) found that virtual contact was not associated with the same mental health benefits as face-to-face, inter-household contact. While those who are remote working are likely to have virtual contact with people, there may be qualitative differences between virtual and face-to-face contact.

*Hypothesis 3. Remote working reduces social contact which is associated with higher levels of the symptoms of depression for women*.

### Changing Levels of Exercise

The final potential mechanism that we consider is levels of exercise. The link between positive mental wellbeing and physical activity and exercise are well-known (Donaghy, 2007; Schuch et al., 2018) and exercise has been associated with levels of mental ill-health during the COVID-19 pandemic (Pieh et al., 2020; Rogers et al., 2020; Cecchini et al., 2021). However, the association between social distancing and levels of exercise and physical activity during the pandemic is variable. Some countries have had strict rules about outdoor exercise particularly, e.g., Italy banned it for a period of time in 2020. There are claims that physical activity levels have been maintained or increased (Spence et al., 2020), but also that they have declined (Violant-Holz et al., 2020). In Great Britain, levels of exercise are reported to have increased among people in higher income bands (Office for National Statistics, 2020c).

On the one hand, working from home has the potential to reduce the time spent commuting and may therefore mean that people feel they have more time to dedicate to exercise. There may also be a growing awareness of the value of exercise to mental wellbeing. For example, government rules about social distancing in the UK highlighted that people could leave their house to exercise for one hour a day, and the public communications about the rule extolled the benefits of exercise for mental wellbeing. On the other hand, the closure of gyms and other recreational spaces has disrupted routines and this may mean that exercise had to become a more purposeful activity, which relies on higher levels of motivation (Spence et al., 2020). Furthermore, people's commute is often a major component of their daily physical activity (Rafferty et al., 2016) and working from home has been associated with an increase in sedentary behavior (McDowell et al., 2020) so stay at home orders seem likely to result in reduced physical activity. Women particularly have reported less intensive physical activity during the initial stages of the pandemic (Rogers et al., 2020). The literature demonstrates that the effect of working from home on levels of exercise is uncertain, demonstrating the relevance of exploring this variable within our study.

*Hypothesis 4a. Remote working reduces levels of exercise which is associated with higher levels of the symptoms of depression for women. || Hypothesis 4b. Remote working increases levels of exercise which is associated with lower levels of the symptoms of depression for women*.

**Figure 1:**
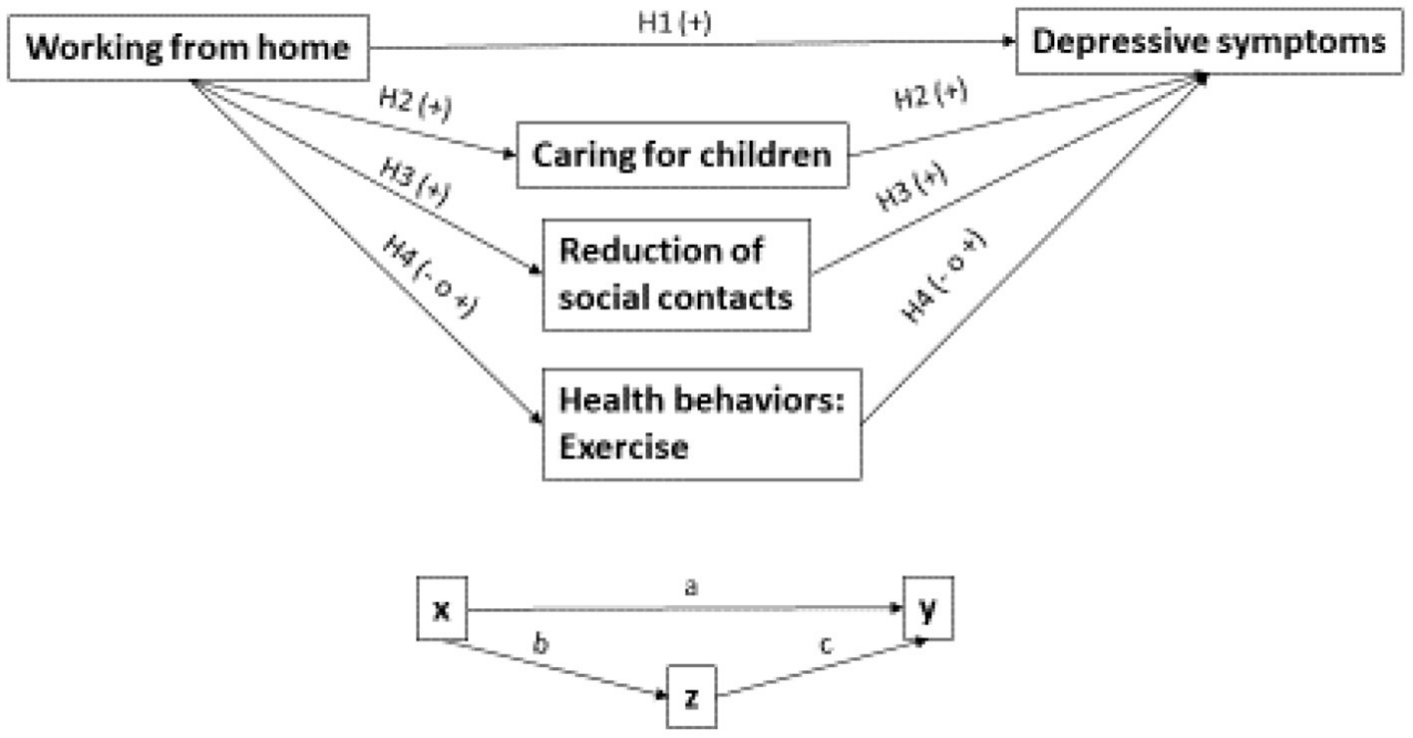
Causal model for mediation mechanisms.

Figure 1 summarizes our causal model for mediation mechanisms.

## Data and Variables

To test the suggested hypotheses, we apply linear regression modeling techniques to a non-probability sample obtained from the “Pandemic Emergency in Social Perspective: Evidence from a large Web-survey research” (PESP-6) study. PESP-6 is a cross-sectional study in which a total of 11,340 respondents, living in six European countries (Italy, Sweden, United Kingdom, France, Poland, and Czech Republic) completed a survey between March and July 2020. We included data from respondents who have completed at least 85% of the questionnaire (total 9,541 observations). Moreover, on inspecting the data, we found that the sample from Czech Republic was considerably older in comparison to the other countries, which limited the number of working-age women in the sample. Therefore, we did not include data from Czech Republic and our analysis considered data from five countries: Italy, Sweden, United Kingdom, France, and Poland (total 7,991 observations). We choose these countries because they differ on various dimensions. Firstly, they belong to different geographical areas (Northern Europe: Sweden and the UK; Western Europe: France; Eastern Europe: Poland; Southern Europe: Italy); secondly, they adopted different approaches to manage the initial stage of the COVID-19 pandemic (more restrictive in Italy, France, and the UK, less restrictive in Sweden and Poland) (Webb et al., 2021). Moreover, we relied on local researchers to collect data. Although data were collected during different time periods in each country, fieldwork started after social distancing measures, in particular, workplace measures and the closure of educational institutions were enforced or recommended (see Table 1). All countries introduced compulsory social distancing measures, with the exception of Sweden where social distancing was encouraged but not mandated. In Sweden, people were urged to work from home but primary schools remained open with secondary and higher education institutions advised to implement remote teaching (COVID-19 Health System Response Monitor, 2020).

**Table 1:** Time line of social distancing measures and survey by country.

**Country**	**Time-spell of survey**	**Workplace measures (i.e., teleworking enforced except for essential workers)**	**Closure of educational institutions (i.e., child care, primary, secondary and high education schools)**	**“Stay-home” order or recommendation**
IT	29 March−14 April	12 March−10 June	4 March−14 September	10 March−4 May
FR	13 April−15 June	17 March−10 May	16 March−22 June	17 March−2 June
PL	15 April−12 June	8 March–n.a.	12 March−30 June	24 March−19 April
UK	09 June−07 July	20 March−9 May	18 March−1 September	23 March−10 May
SW	18 May−09 July	6 March−30 September (recommended)	No closure	No lockdown

*Data Source for IT, FR, PL, SW: European Centre for Disease Prevention Control (ECDC) the Joint Research Centre (JRC) of the European Commission (2021)*.

*Data Source UK: European Centre for Disease Prevention Control (2021) (accessed: October 28, 2021)*.

The PESP-6 questionnaire comprised of 31 questions relating to the social and psychological impact of the COVID-19 pandemic and captured whether respondents had changed their behavior and lifestyle practices during the pandemic. The first version of the questionnaire was designed in Italian and subsequently translated by the researchers of each country involved in the study.

Five cognitive interviews based on the Italian language version of the survey took place prior to the administration of the questionnaire. These interviews tested whether respondents interpreted the item wording and answer scale design as intended and these interviews informed the improvements made to the questionnaire. Cognitive interviewing helps to increase internal validity by confirming comprehensibility and identifying question wording that needs to be clarified (Campanelli, 2008). The questionnaire was administered by the PESP-6 study team online using the Qualtrics (2020) software package and was distributed via social media in order to achieve a large, convenience sample. A link to the survey was distributed via Facebook, Twitter and WhatsApp groups. PESP-6 did not purchase advertising to distribute the link.

In view of our interest in the experiences of women working from home, we restrict our analysis to women (total 6,242 observations) aged between 25 and 65 years old (total 5,335 observations), working either at their usual workplace or from home, also referred to as smart working/remote working). The sample was limited to women aged 25 years and older because, across the European Union, the mean age of women giving birth to their first child is 29 years (although there is variation across countries) (Eurostat, 2021). Therefore, it was expected that this group would be more likely to experience conflict between caring responsibilities and employment than a younger sample aged 18 years and older. Working was self-defined as those who were both employed and self-employed and did not distinguish between part and full-time work. In line with this, across the 5 countries (France, Sweden, UK, Italy, and Poland), a total number of observations of 2,865 was achieved. Despite the fact that interesting research questions could be answered from a cross-country comparison—such as, investigating the mechanisms between working from home and depression in different contexts—limits in our dataset (e.g., lack of data on paid and unpaid work within couples) restricted the exploration of comparison. Therefore, the sample was pooled by country, opting for a simpler strategy of maximizing statistical power. Finally, women who reported chronic diseases or long-lasting health problems, and women with serious limitation in daily activities were further excluded from the sample in order to limit potential health selection biases, i.e., reverse causality. This left a sample of 2,771 observations.

Finally, we used listwise deletion in the analysis to respond to missing values. Cases that have missing values in at least one of the variables of interest—namely the variable used in our models—were deleted. As said, after the first selection of the sample by country, age, gender, working status and health impairments, we obtained 2,771 observations. Among these, cases with missing values represented 4% of the sample (*N* = 112). The final analytical sample included 2,659 observations (see Table 2 for a description).

**Table 2:** Description of the sample and distributions.

	**Working at the workplace** **40.00%**	**Working from home** **60.00%**
**Country (%)**		
Italy	31.60	68.40
France	38.01	61.99
Poland	40.05	59.95
UK	37.45	62.55
Sweden	59.54	40.46
**Depression (mean PHQ-8 score)**	5.95 (5.28)	6.56 (4.76)
**Age classes (%)**		
25–34	23.16	22.04
35–44	34.84	43.21
45–54	26.55	25.55
55–65	15.44	9,20
**Education (%)**		
Low	10.92	2.07
Medium	29.10	26.86
High	59.98	71.07
**Marital status (%)**		
Not partnered	27.78	24.86
Partnered	72.22	75.14
**Fear of transmitting COVID-19**	5.93 (3.03)	5.60 (2.84)
**Size of residential area (%)**		
**City and large city**	21.66	28.87
Town	51.60	51.91
Village	26.74	19.22
**Caring for children (Children < 14 yrs) (%)**		
No kids	32.02	32.12
Kids <14	46.14	52.22
Kids > 14	21.85	15.19
**Reduction in social contact**	0.87 (0.12)	0.90 (0.11)
**Exercise (%)**	52.82	39.57
No		
Yes	47.18	60.43
**Total *N***	**1,062**	**1,597**

### Main Variables

Our dependent variable *Symptoms of depression* was surveyed using the self-reported 8-item Patient Health Questionnaire (PHQ-8) (Kroenke et al., 2001). The PHQ-8 is internationally established and is a valid and reliable diagnostic and severity measure for depressive disorders in large clinical and population studies (Kroenke et al., 2001, 2009). The eight items measure the severity of depressive symptoms over the last 2 weeks on a '4-point' scale, ranging from 0 (not at all) to 3 (nearly every day). Examples of statements incorporated in the PHQ-8 include: “little interest or pleasure in doing things” and “feeling down, depressed, or hopeless”. The scores are summed to produce a scale score ranging 0 to 24, with higher scores indicating greater severity of depression. Our sample of working women report an average of 6.32 points on the PHQ-8 scale (st.dev 4.99), which do not vary greatly across countries (5.92–7.03).

Our main independent variable *Working from home* identifies respondents who were smart/remote working during the lockdown. The five countries used different terms for working from home and the questionnaires reflected these differences. The most common term was used and, if necessary, synonyms included in parentheses. For example, the English language version used the term “smartworking/remote working”, whereas the Italian version referred to “smartworking (lavoro agile, lavoro in remoto, lavoro a distanza, da casa)”. To account for the fact that our data stem from a non-probability sample and in order to reduce the bias in the distribution of socio-demographic and contextual characteristics in our sample, we adjust our multivariate regressions with a broad range of covariates: *age classes, level of education, marital status, fear of transmitting COVID-19 to others, size of area of residence (number. of inhabitants), time of the interview and country*.

### Mediators

During periods of social distancing, the closure of schools and restrictions on extracurricular activities meant that many children's activities were transferred from outside to inside the home. Working parents experienced greater demands on their time in order to meet their children's care and educational needs. This was especially the case for parents with young children. The variable “children under 14 living at home” was used to establish the association between increased time pressures due to childcare and the prevalence of symptoms of depression.

In order to measure how and which social contacts changed during periods of social distancing, the survey asked respondents to report the frequency (less frequent, same frequency, more frequent) of face-to-face contact with: 1. Close family members; 2. Non-cohabiting relatives; 3. Friends; 4. Work colleagues; 5. Neighbors. On the basis of this 5-item measure, we built an additive index which was rescaled to between 0 and 1, where values close to 1 meant less frequent face-to-face interaction. Thus, our index measured “reduction in social contacts”.

Among other health behaviors, PESP-6 asked people to report whether they were exercising during these early stages of the COVID-19 pandemic. Four responses were available: 1. No; 2. Yes, with the same frequency as before (or more); 3. Yes, although less frequently; 4. Yes, I've started to do it since I've been in the social distancing period. The variable is recoded as binary, with the value 0 = “Not doing exercise” and the value 1 = “Yes, doing exercise” (the three positive answers of the original variable are collapsed into the same category). We were interested in exploring the association between depression and participating in any amount of exercise and so we did not distinguish between the amount of exercise people were undertaking.

### Strategy

As the PHQ-8 measure runs on a 0–24 scale, we estimate standard OLS linear models to test our hypothesis.

Moreover, we test the three proposed mechanisms by employing mediation analysis and compare Model 3 through Model 5. In order to decompose the total effect of *Working from home* into direct and indirect effects, we need to compare the estimated coefficient of our main independent variable (IV) between a reduced model without the mediating variables (MV) and a full model with one or more mediating variables added.

In this article, the decomposition of the “total effect” into “direct and indirect effect” is done by using the method developed by Breen et al. (2013) for mediation analysis (further KHB method). Notably, the KHB method was originally developed for comparing coefficients across nested non-linear models (Breen et al., 2013) but it is also suitable for other models in the generalized linear model family (Kohler et al., 2011). The KHB method is particularly suitable for our research question on mediation mechanisms, as it recovers the degree to which a third variable, Z, mediates or explains the relationship between X and the outcome variable, Y (see Figure 1). Most importantly, it also provides analytically derived statistical tests (Kohler et al., 2011) for direct and indirect effects, which would not be otherwise available via only regression models, and therefore allows us to test our hypothesis on mediation mechanisms.

## Results

In order to test our first hypothesis, we implement two regression models. Table 3 shows the linear coefficients (not standardized) for our main independent variable and covariates, namely the mean increase or decrease of depression (DV) for working from home, net of other variables. Full models are available on reasonable request to the authors.

**Table 3:** Linear regression models.

	**M1**	**M2**
**Home-working**	0.57^***^	0.65^***^
	(0.20)	(0.20)
**Constant**	5.80^***^	6.03^***^
	(0.22)	(0.48)
***N***	2,659	2,659
**R-squared**	0.022	0.091

*Robust standard errors in parentheses*.

*** *p < 0.01*,

^**^p < 0.05,

^*^p < 0.1.

*M1 adjusted for country dummies, date of interview*.

*M2 adjusted for age classes, education, marital status, size of residential area, fear of transmitting COVID, country dummies, date of interview*.

*Depression in working women. PESP-6 (UK, PL, IT, FR, SE). NOT standardized beta*.

Model 1 represents our baseline—the association between homeworking and the symptoms of depression, only adjusting for country and date of interview. The relationship is positive and highly significant: working from home increases depression by 0.57 points on average compared to women who were working from their usual workplace. In substantial terms this is an increase of almost 9% in the prevalence of the symptoms of depression [(0.57/9.80)^*^100]. Model 2 adds individual variables (i.e., age classes, education, marital status and fear of transmitting COVID-19) and contextual variable (i.e., size of residential area) as covariates. Here, the coefficient for working from home increases and is still positive and highly significant. Net of other variables, women who work from home experience higher symptoms of depression than women who were working in their usual place of work, with a difference of 0.65 points on the PHQ-8 scale. This equates to almost an 11% increase in depressive symptoms. Overall, our results confirm our first hypothesis. In our data, women working from home during the initial months of the COVID-19 pandemic experience more symptoms of depression than those who remained working at their usual place of work.

Table 4 shows the coefficients obtained by three KHB models. First, to test our second hypothesis, we decompose the effect of *Working from home* on *Depression* by using *Caring for children*. Model 3 shows a null and not significant indirect effect of *Caring for children* (0.00). Our second hypothesis is rejected as a result. *Caring for children* does not appear to explain the prevalence of the symptoms of depression for women working from home.

**Table 4:** KHB models based on linear regressions.

	**M3**	**M4**	**M5**
Total effect	0.65^***^	0.65^***^	0.65^***^
Direct effect	0.65^***^	0.59^***^	0.72^***^
**Indirect effect**			
*via* Caring for children	0.00		
*via* Reduction of social contacts		0.06^*^	
*via* Exercise			−0.07^***^

*Decomposition of Total Effect of Working from home on Depression into Direct Effect and Indirect Effect- PESP-6 (UK, PL, IT, FR, SE)*.

Second, we include *Reduction of social contacts* to the decomposition to test our third hypothesis. In Model 4, the total effect of *Working from home* (0.65^***^) is decomposed in the direct effect (0.59^***^) and the indirect effect of *Reduction of social contact* (0.06^*^), indicating a small and significant mediation effect of *Reduction of social contact*. Our third hypothesis is confirmed: although slightly, working from home affects symptoms of depression via reduced social contact.

We repeat the procedure by adding *Exercise* (Model 5). *Doing exercise* (−0.07^***^) mediates the relationship between *Working from home* and *Depression*. As the indirect effect via *Exercise* has a negative sign and exercise is negatively correlated with depression (full models are available on reasonable request to the authors), it means that the causal path follows our hypothesis 4b. *Remote working increases levels of exercise which is associated with lower levels of depression for women*.

## Discussion

### Summary of Key Findings

The study has demonstrated that, for women during the initial stages of the COVID-19 pandemic, working from home is associated with a greater prevalence of the symptoms of depression compared to those women who continue to go to their usual place of work. Remote working entails the blurring of the boundaries between the workplace and the home (Chung et al., 2020). While it is clear that working mothers were bearing the brunt of increased domestic demands created by homeschooling and the closure of child care facilities due to social distancing measures (Chung et al., 2020; Farré et al., 2020; Hipp and Bünning, 2021), our study suggests that having a child under the age of 14 at home does not in itself significantly increase depressive symptoms. This stands in contrast with other literature which has linked the experience of poor mental health in lockdown with the increased demands of childcare (Benassi et al., 2020; Zamarro and Prados, 2020). Our findings suggest that during periods of social distancing, the increased pressures likely experienced by remote working women with children aged 14 years or younger may not necessarily result in the higher prevalence of symptoms of depression.

Instead, it would appear that reduced social contact is associated with higher levels of depression for women working from home, whether they have children or not. Studies have shown that for those experiencing loneliness, this perception appeared to increase as social distancing continued (Bu et al., 2020). When discussing their findings on the association between remote working and satisfaction with work and family life, Möhring et al. (2021, p. S613) question whether their findings are generalizable and note that the effect of working from home on wellbeing may be different if social contacts are maintained. Although we recognize the distinction between the subjective experience of loneliness and social isolation, our study adds to this literature by demonstrating that when working from home also involves reduced face-to-face social contact with family, friends and colleagues, this social isolation can lead to increasing symptoms of depression.

Our study also shows that, for women, exercise appears to offer some protection against depressive symptoms, and this finding adds to evidence on the general importance of exercising for mental wellbeing. Previous studies have shown that women were less likely to be physically active during the pandemic (Rogers et al., 2020) and the gendered aspects of exercise discussed in the literature are vital to understand in pandemic conditions.

### Limitations

While this study makes a valuable contribution to understanding of the association between COVID-19 lockdowns and levels of depression in women, there are several limitations. Our sample is self-selecting and so those who responded may not represent the range of experiences within the wider population. Efforts to recruit respondents often relied on the use of social media, consequently, women who are not part of these networks may be inadvertently excluded within the sample. This is of particular concern given the focus of this study on the association between reduced social contact and the prevalence of the symptoms of depression. It may be that people who do connect with others via online channels may have experienced social distancing differently. The reduced social contact resulting from social distancing may have had an even greater impact on mental wellbeing in this group. Because of selectivity of our sample, our findings are of limited generalizability (Kohler et al., 2019).

Secondly, limitations of the PESP-6 dataset contributed to a number of weaknesses within our analysis. The cross-sectional nature of the data and the lack of information on levels of the symptoms of depression prior to the COVID-19 pandemic make it difficult to totally account for reverse causality within our analysis. Nevertheless, we believe that excluding from the sample women with disabilities and long-term health problems allowed us to limit this potential selection bias. For the same data-related reasons, we could not fully control for observed and unobserved heterogeneity, despite the fact that our estimates are adjusted for a broad range of individual and contextual factors. While it would appear that there are significant associations between working from home and the experience of the symptoms of depression, our confidence in this finding would be increased if we could call upon longitudinal data in order to explore whether initial lockdowns led to an increase in the symptoms of depression.

Moreover, our study tested the association between working from home, the demands of providing childcare and the prevalence of the symptoms of depression. This variable assumes that women working from home are experiencing greater domestic demands than prior to the introduction of social distancing measures. Unfortunately, we do not have data on how they and any partner living at home divide domestic duties, or (perhaps of greater relevance for mental wellbeing) how women perceive their dual roles as employee and parent (Oster and Scannell, 1999). The dataset also does not include any measure of the number of hours worked by respondents' partners, or respondents, which would have developed our analysis of the demands of providing childcare and, when used as control, make our results more robust. Our analysis could have been further developed by considering whether the experience of single mothers is different from that of co-habiting couples or co-parents. We were also not able to assess other caring responsibilities, such as caring for elderly or otherwise vulnerable adults. Given the relatively large impact of COVID-19 on older and otherwise vulnerable populations, the mental health impact of caring for these groups may have been significant. The dataset did not have a measure for the subjective experience of loneliness. Therefore, we are unable to distinguish between social isolation and perceived loneliness. However, given the vast exogenous shock presented by the start of the COVID-19 pandemic, it perhaps could be assumed that reduced social contact would lead to increased loneliness. Nevertheless, we could not include perceptions of loneliness within our analysis.

Further, our analysis does not make comparisons across countries and so we do not know the extent to which the relationship between working from home and depression and its mechanisms work within each country. The countries were selected by the PESP-6 team to reflect a geographical distribution which includes Northern, Central and Southern European countries. Although we recognize that would have been interesting to analyze cross-country differences in how and why working from home relates to depression, limits in the PESP-6 questionnaire did not allow us to pursue this research path (e.g., lack of detailed information about women's working conditions and arrangements). Rather, we focused in exploring women's experience of homeworking at a broader level and reducing the legal and cultural contextual variance by controlling for country dummies. Nevertheless, we believe that additional research is needed to explore the relationship between homeworking during the early stages of the pandemic and the symptoms of depression on a country basis.

A final limitation is that the data analyzed in this study were collected at the relatively early stages of the pandemic. The associations between working from home and the symptoms of depression may have changed as the pandemic has progressed through subsequent waves and the reintroduction of associated social distancing measures, as well as changing political and economic pressures.

### Further Research

There remains a great deal of uncertainty regarding the trajectory of the COVID-19 pandemic. However, as public health programs such as vaccination are implemented and anti-viral treatments for COVID-19 improve, policy discussions and public debate are turning to exploring how we may live with the virus in future and/or face potential variants or other viral pandemics. Understanding changes in working practices, new forms of social network support building and understanding more about the effects of remote working on mental wellbeing should be an aim for future research. In particular, there is scope to develop the longitudinal approaches as seen in studies by Möhring et al. (2021), Cecchini et al. (2021), and Niedzwiedz et al. (2021) to explore wider trends in mental ill-health throughout, and after, the duration of official “lockdowns” and wider social distancing measures.

As increased rates of remote working are likely to persist, even after the pandemic, it is necessary to explore further how the benefits of reduced commuting and potential greater autonomy can be harnessed while mitigating risks such as reduced social contact. During the early stages of the COVID-19 pandemic, there are likely to be context-specific factors which are likely to be associated with the increased prevalence of mental health problems and these variables are unlikely to continue after the pandemic. While people working from home will have different personal circumstances, our findings indicate that those who experience reduced social contact may be more likely to experience the symptoms of depression. Exploring the differential effects of reduced social contact on people of different genders in greater detail will add to our understanding of the gendered gap in mental health, for instance, whether it the case that women have larger social circles, meaning that they have “more to lose” from social distancing (Etheridge and Spantig, 2020) or whether people of different genders experience reduced social contact in different ways. Maestripieri (2021) calls for greater recognition of intersectionality when studying the social and economic implications of COVID-19. In addition, it would be useful to break down our understanding of reduced social contact through other, intersectional lines of difference, such as ethnicity and class.

Given the positive association between mental wellbeing and physical activity, examining some of the barriers preventing women from exercising during periods of social distancing may have significant implications for policy. Furthermore, it would be interesting to explore whether this association still stands as many countries face second and third waves of the pandemic with associated social distancing measures. There are some indications that, in Northern European climates, exercise may have decreased during winter months, with women reporting lower levels of exercise (Fancourt et al., 2020) which may have had a further effect on women's experience of working from home and the potential to challenge the gender gap within mental wellbeing.

While we did not see an association between caring for children and depression, our study does not include data on women's perceptions about how COVID-19 lockdown is affecting their levels of productivity or feelings of job satisfaction (Feng and Savani, 2020). If available, incorporating these data would strengthen our operationalization of the potential conflict between employment and caring responsibilities and offer a further avenue of research.

## Conclusion

This study has focused on how place of work may influence the likelihood of experiencing the symptoms of depression. The introduction of policies requiring citizens to socially distance has vastly increased the number of people working from home. Our study has demonstrated that reduced face-to-face social contact while working from home is associated with a higher prevalence of the symptoms of depression. While we note that working from home during a pandemic is likely to be in many ways a different experience compared to “normal” times without social distancing restrictions, the study has relevance both for future pandemics and because of public and policy debates around the likelihood that rates of home-working will remain elevated, even after the COVID-19 pandemic.

This study highlights the importance of maintaining social contact when remote working. While employers may be supportive of increased home-working, there is also a need to consider how to support social connections between colleagues and to ensure time in the working day (particularly during restricted daylight hours) for employees to engage in exercise. Any exploration of the effects of the pandemic on workplace expectations and preferences should be cognisant that women and men with children may have different experiences of working from home. Broadly, women often shoulder the majority of caring responsibilities within the home. Our findings question the association between remote working, caring for children, and depressive symptoms. Nevertheless, if remote working entails a shift toward a more unstructured workday, it may mean that working mothers have less time to devote to maintaining face-to-face social connections or exercise. Employees should also be mindful of the importance of maintaining social connections and maintaining physical activity when working from home. Furthermore, employers should be aware that people are likely to have different experiences of remote working depending on their domestic circumstances.

## Data Availability Statement

The raw data supporting the conclusions of this article will be made available by the authors, without undue reservation.

## Ethics Statement

The studies involving human participants were reviewed and approved by University of Birmingham Social Sciences and Humanities Ethical Review Committee (ERN_20-0642). The patients/participants provided their written informed consent to participate in this study.

## Author Contributions

EB prepared the first draft of the article, conducted a literature review, and co-ordinated the analysis and interpretation of the results. GT conducted the statistical analysis. EB and GT are joint first authors of this article. IW and GT contributed to the interpretation of the results. LL conceived the comparative europe study and co-ordinated the international partners. NG led the UK arm of the study, adapted the research instruments, secured ethical approval for the UK data collection, and contributed to the interpretation of the results. All authors contributed to the article and approved the submitted version.

## Conflict of Interest

The authors declare that the research was conducted in the absence of any commercial or financial relationships that could be construed as a potential conflict of interest.

## Publisher's Note

All claims expressed in this article are solely those of the authors and do not necessarily represent those of their affiliated organizations, or those of the publisher, the editors and the reviewers. Any product that may be evaluated in this article, or claim that may be made by its manufacturer, is not guaranteed or endorsed by the publisher.
